# Effectiveness of acupuncture for breast cancer related lymphedema: protocol for a single-blind, sham-controlled, randomized, multicenter trial

**DOI:** 10.1186/s12906-017-1980-0

**Published:** 2017-09-21

**Authors:** Huiru Zhu, Jinwan Li, Zheng Peng, Yujie Huang, Xiaolan Lv, Liuying Song, Gechen Zhou, Shengzhang Lin, Jifei Chen, Baoyu He, Fengxian Qin, Xumexiang Liu, Meiyu Dai, Yan Zou, Shengming Dai

**Affiliations:** 1 Department of Galactophore, the Third Affiliated Hospital of Guangxi University of Chinese Medicine, Liuzhou, Guangxi China; 2 Department of Clinical Laboratory, the Fourth Affiliated Hospital of Guangxi Medical University, Liuzhou, Guangxi China; 3 Department of Clinical Laboratory, Liuzhou Maternity and Child Health Care Hospital, Liuzhou, Guangxi China

**Keywords:** Acupuncture, Breast cancer related lymphedema, Protocol, Trial

## Abstract

**Background:**

Although various treatments for breast cancer related lymphedema exist, there is still a need for a more effective and convenient approach. Pilot studies and our clinical observations suggested that acupuncture may be a potential option. This study aims to verify the effectiveness of acupuncture on BCRL and evaluate its safety using a rigorously designed trial.

**Methods/Design:**

Women who are clinically diagnosed as unilateral BCRL, with a 10% to 40% increase in volume compared to the unaffected arm, will be recruited. Following baseline assessment, participants will be randomized to either the real acupuncture group or sham-acupuncture group at a ratio of 1:1, and given a standard real acupuncture or sham-acupuncture treatment accordingly on both arms followed by the same usual care of decongestive therapy. Volume measurements of both arms will be performed for every participant after each treatment. Data collected at baseline and the last session will be used to calculate the primary outcome and secondary outcomes. Other data will be exploited for interim analyses and trial monitoring. The primary outcome is the absolute reduced limb volume ratio. Secondary outcomes are incidence of adverse events and change in quality of life. A t test or non-parameter test will be used to compare the difference between two groups, and assess the overall effectiveness of acupuncture using the SPSS software (version 12).

**Discussion:**

This study will help expand our knowledge about the effectiveness of acupuncture on BCRL, and how acupuncture might be used in the management of this condition. Acupuncture may be a promising complement or alternative to conventional lymphedema treatment methods, if its effectiveness is confirmed.

**Trial registration:**

ClinicalTrials.gov NCT02803736 (Registered on October 31, 2016).

**Electronic supplementary material:**

The online version of this article (10.1186/s12906-017-1980-0) contains supplementary material, which is available to authorized users.

## Background

As a long-term complication stemming from the disruption of lymph flow, breast cancer related lymphedema (BCRL) is a distressing problem afflicting around 20% of women who underwent breast cancer treatment, and is characterized by the chronic swelling of one or both upper extremities [1–4]. Depending on the extent of the surgery and auxiliary treatments, BCRL affects patients differently, and may develop one or several years later after surgery [5]. With the increase in breast-cancer incidence as well as the five-year survival rate, it is estimated that there are approximately 530,000 patients facing BCRL in China only [6]. Since patients under this condition often require lifetime management [7], it imposes a big burden on the society with long-term costs, and makes up a significant source of hardship for many families due to health-related productivity loss [8, 9]. According to research, common risk factors for BCRL includes obesity, axillary lymph node dissection, postoperative radiotherapy, infection, and history of lymphangitis [10, 11]. Moreover, there is no cure for BRCL so far, and the only way is to avoid risk factors, reduce the severity of symptoms and improve the function of the affected arm. As a result, almost every patient experiencing breast cancer surgery is advised to take precautions and take good care of the affected arm to prevent lymphedema [12, 13]. All these things pose a reminder to patients of their malignant status and become a prominent psychosocial problem that afflicts breast cancer survivors [14, 15]. As a result, the quality of life is much lower for patients after breast cancer surgery than those without this condition [16–19]. At present, there are various treatment options to deal with BCRL, including lymph drainage, low-level laser, micro-surgery, and transplantation of axillary lymph nodes [20–24]. However, no consensus has been reached on any specific approach for BCRL [25], and the management of BCRL remains a major challenge for both physicians and patients [26].

In the theory of Traditional Chinese Medicine (TCM), diseases like BCRL are considered as the same “zheng” as “edema” [27], and is thought to be caused by qi deficiency and blood stagnation. Acupuncture, as an ancient therapy in TCM, is thought to have the efficacy to stimulate the transformation of qi and drainage of dampness [28], and is widely used for various diseases including edema in East Asia for thousands of years [29]. Previous researches in patients with lymphedema suggest that this treatment might reduce swelling by improving circulation without inducing safety issues [30–35]. However, results regarding its effectiveness in the reduction of swelling have not yet been well established, and no randomized controlled trial has been performed on this issue. Therefore, with this protocol, we aim to evaluate the effectiveness of acupuncture for the treatment of BCRL.

## Method

### Study setting/design

This multi-center two-arm randomized clinical trial will be performed in the following three medical centers: the Fourth Affiliated Hospital of Guangxi Medical University (about 400 breast cancer patients seen annually), the Third Affiliated Hospital of Guangxi University of Chinese Medicine (about 200 breast cancer patients seen annually), and Liuzhou Maternity and Child Health Care Hospital (about 120 breast cancer patients seen annually). A fixed quota (number of participants recruited) will be distributed to each center based on their total number of breast cancer patients yearly (Additional file 1). The recruitment would start from December, 2016, and will end in October, 2019 synchronously in the three centers. For patients assigned to the real acupuncture group, a standard acupuncture procedure using the real will be performed; for patients assigned to the sham acupuncture group, a standard acupuncture procedure using the sham needles will be performed. The acupoints, frequency (3 times a week for 4 weeks), and duration (30 min after de qi) of each patient in both groups should be the same. All participants in both groups will receive the same usual care of decongestive therapy following acupuncture. An overview of the enrollment procedures, the study design, and the assessments of this trial is illustrated in Table 1.

**Table 1:**
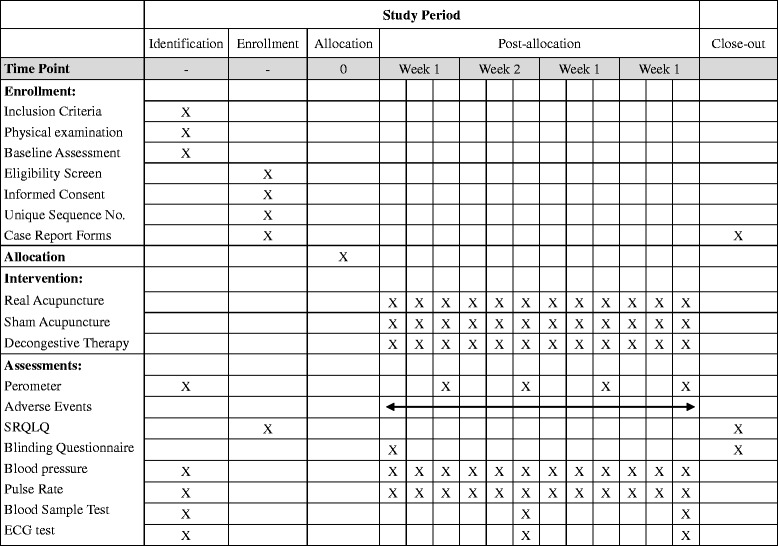
Procedures and time-points of enrollment, intervention, and assessment in this trial

This study has been approved by the Institutional Review Board of the Fourth Affiliated Hospital of Guangxi Medical University (Approval Number: PJK2016090).

### Study oversight

Three committees will be in charge of the supervision in this trial.

1. The trial steering committee (TSC).

The TSC, composed of trial designers of the trial and coordinators of each center, is responsible for key technical and operational problems, such as changes in eligibility criteria, outcomes, analyses, etc. A meeting will be held every month.

2. The trial coordinating committee (TCC)

The TCC, composed of coordinators, acupuncturists and nurses in each center, is in charge of specific business, such as recruitment and identification of possible participants, information dissemination of this trial, collection and preservation of data and reports, and management of trial sites. The coordinators in each center are responsible for the establishment of the standard operation procedures and the training of relevant staff prior to the initiation of this trial. The TCC will cooperate with TSC and make a report every month.

3. The data and safety monitoring board (DSMB)

The DSMB, composed of experienced experts in experimental design and statistics, is responsible for review and evaluation of the trial periodically. They mainly focus on issues such as execution of the trial, collection of the data, participants’ safety, and personal privacy protection. They will also report to the TSC monthly and may provide advice in the modification or termination of the trial.

### Eligibility criteria

In this trial, following inclusion criteria will be adopted:

- ▪ women who have completed breast cancer treatment for more than 1 year;
- ▪ no evidence of recurrence;
- ▪ unilateral lymphedema resulting from surgery for breast cancer;
- ▪ age between 20 and 45;
- ▪ BMI between 18 and 25;
- ▪ mild to moderate lymphedema (10% to 40% increase in volume compared to the unaffected arm).
- Patients will be excluded as follows:
- ▪ bilateral lymphedema;
- ▪ current use of chemotherapy or radiotherapy;
- ▪ history of bilateral axillary lymph node dissection;
- ▪ serious lymphedema, >40% increase in volume compared to the unaffected arm;
- ▪ pregnant women or overweight women;
- ▪ unable to adhere to the protocol or the treatment schedule;
- ▪ recurrent breast cancer or evidence of other active cancer;
- ▪ current use of diuretics like Diosmin, or other investigational drugs;
- ▪ contraindications for acupuncture, such as infections, open wounds, or broken skin.

### Forbidden drugs and concomitant treatments

To avoid cross effect, following drugs are unacceptable in this trial, including Diosmin, Hesperidin, and diuretics. Other concomitant treatments should be consulted with the physician in charge and meticulously recorded.

### Recruitment of participants

The nurses in each center will identify possible participants according to the inclusion and exclusion criteria. Then possible participants will be invited to attend a screening visit, during which a routine physical examination and a baseline assessment including the upper limbs and risks of recurrence will be performed. The coordinators in charge will confirm their eligibility based on their health status and inclusion criteria. For every eligible participant, a unique number will be given by the coordinators based on their enrollment sequence after a written informed consent was obtained, and data on demographic information will be collected through interviews and questionnaires by the acupuncturists funded by this project.

### Randomization and allocation

Eligible participants in each center are randomized into the real acupuncture group and the sham-acupuncture group at a ratio of 1:1 according to the randomization sequence, which is a series of randomization numbers representing each patient’s unique number, and pre-generated by the chief designer using RAND function in Microsoft Excel program according to the quota of each center (Additional file 1).

### Blinding

In this trial, participants and statisticians are blinded to the group assignment, whereas acupuncturists are not, due to the nature of this trial. However, to minimize the unintentional physical cues and bias in this trial, acupuncturists will be required to emulate the same procedure for the sham-acupuncture groups. And the credibility of blinding will be assessed by experiments before the formal trial, and adjustments will be made until it is acceptable. The process of the blinding will be monitored and assessed by the DSMB.

### Intervention

Eligible participants will be required to set up an appointment by the acupuncturist according to their symptoms patterns, such as the time period their lymphedema manifest the most typical symptoms. Upon arrival at scheduled appointments, participants will receive real or sham acupuncture accordingly on both arms. In this trial, we apply sterilized stainless steel needles (0.25 × 40 mm; Asia Med, Munich, Germany) in real acupuncture group, and blunt telescopic placebo needles (Streitberger needle; Asia Med, Munich, Germany) in sham-controlled group. All procedures will be performed by officially registered acupuncturists with clinical experience of 3 years at least.

### Real acupuncture group

**Fig. 1:**
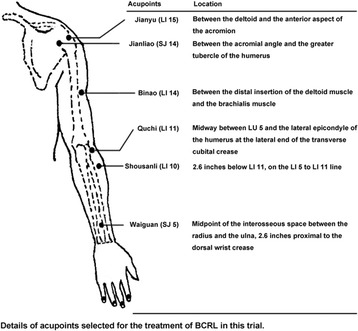
Overview of selected acupoints in this trial. Participants will receive real acupuncture or sham-acupuncture at the same acupoints in both arms 3 times a week for 4 weeks (12 sessions)

Patients in the real acupuncture group will receive true acupuncture 3 times a week for 4 weeks (12 sessions) at 6 acupoints in each arm: Jianyu (LI 15), Jianliao (SJ 14), Binao (LI 14), Quchi (LI 11), Shousanli (LI 10), and Waiguan (SJ 5). These acupuncture points were selected on the basis of classic TCM record, the published literature [30–35], and the consensus between our experienced acupuncturists. Acupoints are detailed in Fig. 1. After the application of alcohol swabs, these acupoints will be covered by adhesive plaster with plastic rings, and stimulated by manual rotation of the needles accompanied by lifting and thrusting gently to make patients get de qi, which is a sensation of aching or distension or numbness elicited by acupoint stimulation [36, 37]. Then needles will stay put for 30 min.

### Sham-acupuncture group

**Fig. 2:**
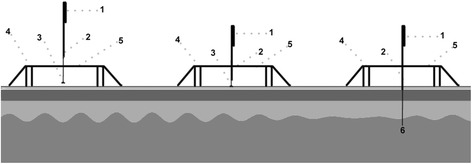
Structure of Streitberger needle. (1) Needle handle (2) Needle rod (3) Blunt tip of the placebo needle (4) Plastic ring (5) Plastic cover (6) Sharp tip of the real needle

Patients in the sham-acupuncture group will receive the same sessions of treatment using Streitberger needles at the same acupoints. Streitberger needles have the appearance of the real needles, however, they will not penetrate the skin. They can be placed at the same acupoints as the real acupuncture group, and held in position by adhesive plaster with a plastic ring, which is conducive to the blinding purpose [38]. When the needle is pushed against the skin, a pricking sensation just like the real insertion will be generated. However, as the pressure increases, the needle shaft will bounce back into the handle, which guarantees the skin unbroken and makes an artificial impression of needle insertion (Fig. 2). Other procedures will be the same with the real acupuncture group, including the application of adhesive plaster and plastic rings.

### Decongestive therapy

For ethical considerations (the interest of participants in sham-group), every participant in both groups will receive the same usual care of decongestive therapy after treatment, which includes a daily manual lymph drainage, gentle exercise, applying moisturizer, and wearing elastic compression garments. Table 2 details the duration, frequency, executor, and location of each step. For participants who take other practice, detailed reports will be required.

**Table 2:** Overview of standard decongestive therapy contents

Standard decongestive therapy	Duration	Frequency	Location	Record	Note
Manual Lymph Drainage	30 min	Daily	Hospital	Massage Therapist	Meticulous records are needed otherwise.
Applying Moisturizer	3 times	Daily	Home	Participant	
Gentle Exercise	2 h	Daily	Home	Participant	
Wearing Compression Garment	3 h	Daily	Home	Participant	

### Patient health monitoring

For each participant in both groups, blood pressure and pulse rate will be monitored following every treatment session, whereas routine blood test, ECG test, renal function test and liver function test will be conducted every 2 weeks (Table 1). These data will be monitored closely by DSMB during the trial.

Moreover, as cuts and other breakage of the skin would incur infection and the deterioration of lymphedema, all participants will be warned. For those developing sudden onset of infection, the treatment should be discontinued till the patients have fully recovered and the coordinator in charge should be contacted as soon as possible. In other cases like serious complications and occurrence of other emergencies, where patients are unfit for further therapies, they will be persuaded to quit.

### Data collection

Before the first treatment session, every participant will get an assessment of both limbs by perometer, which is an automated infrared light digital scanner equipped with a microcomputer. When the limb is placed inside, the infrared light transmitters located on two sides of the perometer will be activated, and the blockage of the transmission of infrared light from both sides caused by the limb will be recorded and calculated. As the transmitters move along the limb, a series of images will be recorded. Hence a highly accurate measurement of the limb size and volume will be created. And these results will serve as the baseline data. Following the treatment of each session, participants in both groups will be asked to take assessments to get the interim data. Upon the last session, the assessments of both limbs will be adopted as the end-point data. For each arm, the measurement will repeat 3 times to ensure the reliability. Table 1 illustrates all the time points when measurements are taken during the whole procedure.

### Primary outcome

#### Absolute reduced limb volume ratio (ARLVR)

The ARLVR is the proportion of absolute reduced limb volume in the affected arm after the therapy, compared with the absolute excess volume in the affected arm before the therapy. This index is inspired by the methods established by Anderson et al. [39] With all data collected at the end of the trial, the ARLVR will be calculated using the following formula:

$$ \mathrm{ARLVR}\ \left(\%\right)=\frac{\left({\mathrm{V}}_{\mathrm{AB}}\hbox{-} {\mathrm{V}}_{\mathrm{AL}}\right)\hbox{-} \left({\mathrm{V}}_{\mathrm{UB}}\hbox{-} {\mathrm{V}}_{\mathrm{UL}}\right)}{\left({\mathrm{V}}_{\mathrm{AB}}\hbox{-} {\mathrm{V}}_{\mathrm{AL}}\right)}\times 100\% $$

In particular, V_AB_ represents the volume of affected arm with lymphedema at baseline; V_AL_ represents the volume of affected arm with lymphedema at the last session; V_UB_ represents the volume of unaffected arm at baseline; V_UL_ represents the volume of unaffected arm at the last session.

### Secondary outcomes

#### Incidence of adverse events (IAE)

The IAE will include every adverse event during the treatment, unless it is confirmed otherwise. Possible adverse events include infection and exacerbation of lymphedema. Participants will be asked to report any significant changes to their health state following treatment. Once adverse event occurs, it should be closely monitored and meticulously recorded until stabilization or resolution, and the chief designer and the coordinators in charge will be informed immediately. Together with the acupuncturist in charge, they will consult on the case, take proper actions and evaluate the severity. Unless it has been confirmed irrelevant with the intervention of this trial, every possible case should be reported timely to the Institutional Review Board. The chief designer is responsible for the report of all adverse events. The DSMB will inspect periodically to ensure that all adverse events are handled properly.

#### Quality of life

Quality of life is assessed using a questionnaire from Patient-Reported Outcomes Measurement Information System (PROMIS; http://www.healthmeasures.net/index.php), which requires participants to evaluate their overall health through 10 self-reported global health items representing physical health, pain, fatigue, mental health and social health. Global Physical Health (GPH) and Global Mental Health (GMH) scales will be computed accordingly, and higher scores reflect better life quality. This scoring system has demonstrated remarkable internal consistency and reliability [40].

### Sample size calculation

As no two-arm, randomized, multi-centered, sham-controlled trial has been performed on this topic, no existing data regarding the sample size are available. However, based on our preliminary clinical data collected and a similar study [41], we assume that the mean and SD of ARLVR in the real-acupuncture group and sham-acupuncture group are (0.32, 0.25) and (0.22, 0.2), respectively, and after calculation using G-Power (V.3.1), the effect size (ES) is 0.4417261. Next we input the parameters above, set the alpha risk (α, type 1 error risk) and beta risk (β, type II error risk) at 0.05 and 0.2 respectively, and choose “t test from two independent groups” and “A Priori: Compute required sample size given α, power and effect size”, then after calculating, the required sample size is 164 (Additional file 2). Assuming the loss to follow-up rate is 20%, then 197 is the total minimum sample size required in this trial. However, to increase the power and make full preparation, we decided to recruit 200 participants, 100 for each arm in this trial.

### Loss to follow-up and data management

In practice, however, participants might withdraw from the treatment program at any time. In that case, a call from the acupuncturist in charge will be given to the participant, and appropriate advice and assistance will be provided. If a participant insists on withdrawing from this trial, an instant evaluation of lymphedema status will be made, if it is possible. Complete case reports and case reports on loss-to-follow-up of the participants will be reported to the coordinators in charge every month. First-hand data will be recorded by the nurse and the acupuncturist. The coordinator in each center will collect and preserve the data every two weeks. When the trial is done, the aggregated data will be extracted by two independent researchers to guarantee the accuracy, and finally submitted to the principal investigator for analysis. During the whole process, the privacy of research participants will be fully protected.

### Statistical and data analysis

Upon the receipt of the final data, the Kolmogorov-Smirnov test (K-S test) will be performed, followed by a test for homogeneity of variance to see whether the variances are equal between groups. If the data distribution fits a normal distribution with equal variance, the independent t test will be employed; otherwise, nonparametric test will be chosen to determine whether there is significant difference between the real acupuncture group and the sham acupuncture group. Moreover, one-way ANOVA will be performed to see whether there is significant difference between three centers. Analysis of covariance adjusted for clinical center and baseline will be applied on the condition that significant difference exists between three centers. In addition, regression analysis will be performed to analyze the effects of co-variances (risk factors) such as BMI, axillary lymph node dissection, postoperative radiotherapy, infection, and history of lymphangitis on the overall outcome of this treatment. A type I level error < 0.05 will be interpreted as statistically significant and 95% CI, mean, and SD will be calculated. Data analysis in this trial will consist of an intent-to-treat (ITT) analysis, a per-protocol (PP) analysis and an interim analysis. The ITT analysis will collect data from all the participants in this trial, and for those loss to follow-up, the last observation carried forward method will be implemented. As for the PP analysis, data collection will be rigorous, and only those participants who strictly comply with the protocol are eligible. The interim analysis in this trial performed by DSMB, will use the interim data collected during the trial to actively monitor the operating status of the trial and help find problems timely. All statistical analyses will be conducted using the SPSS software (version 12).

## Discussion

BCRL can impose great burden on the society and cause significant physical and psychological morbidity to patients [42]. Despite the wide application of less invasive surgical techniques, risk for the development of BCRL still remains [43]. Current treatments options for BCRL are either expensive or rather time consuming [44]. Traditional acupuncture emerges as an efficacious, economical, and convenient choice to manage this chronic condition with few side effects, according to several pilot reports [30–35] and our clinical observation. However, after a thorough search in Pubmed and Embase for relevant articles, we found existing studies on this issue were case reports, small single-arm studies [30–33], or parallel studies using regular routine management or Diosmin as a comparison [34, 35], which cannot rule out the placebo effect and needs to be confirmed by rigorously-designed, large-scale studies. The efficacy of real acupuncture over sham acupuncture in dealing BCRL has not been fully illustrated. Therefore, additional research is required to confirm the efficacy and cost-effectiveness of acupuncture on BCRL.In this study, we will adopt Streitberger needle as the sham-acupuncture instrument. Compared with other methods such as placebo points acupuncture, shallow acupuncture at real acupoints and shallow acupuncture at placebo points, Streitberger needle have significant advantages, which make it a perfect choice to keep consistency and credibility, and avoid possible efficacy brought by placebo point acupuncture or shallow acupuncture. This method has been used in several studies with good performance, and was recommended as a reliable sham control [45–47]. Another merit of this study is the measurement instrument we choose. As pointed out above, perometer can easily and swiftly measure the volume of a participants’ limb with high reliability and minimal inter-operator variability compared with other conventional methods, such as volumeters (using a water displacement tank to measure volume) and tape measures (using a tape to measure limb circumference) [48–50], although the measurements from different methods are not interchangeable [51]. According to the data collected from patients and previous reports, the day to day variability in limb volumes is around 0.5 [52]. What is more, to better embody the real effect of acupuncture on BCRL, we use the ARLVR, a more reasonable and practical index that is calculated based on the surplus between the affected arm and the contralateral arm. Theoretically, it can exclude the interfering factors such as base effect and weight fluctuation.

However, there are some limitations in this study. First, to maximally rule out the placebo effect and subjective bias, a standardized acupoint regimen is applied in this trial, which is not in accordance with the concept of individualized therapy of CTM. And additional studies are needed to determine the difference between the standardized acupoint regimen and the individualized acupoint regime. Second, according to TCM, de qi, the needle sensation during acupuncture, is regarded as an important sign of successful acupuncture, and is essential for exerting the clinical efficacy of acupuncture. However, for blinding purpose, acupuncturist are forbidden to enquire about it in both groups in this trial. Finally, as acupuncture is common approach in dealing various symptoms in China, chances are good that some participants in the sham-acupuncture group may feel the difference. Double blindness is not applied in this study, given that the procedure is conducted by the acupuncturists.

In conclusion, this article presents a rigorously designed trial to verify the efficacy of acupuncture on BCRL. The results of this trial will provide more information on this issue.

## Additional file

Additional file 1: Block Randomized Sequence. (PDF 83 kb)
Additional file 2: Sample Size Calculation and G Power Manual. (PDF 65 kb)

## Abbreviations

ARLVR: Absolute reduced limb volume ratio
BCRL: Breast cancer related lymphedema
CI: Confidential interval
DSMB: Data and Safety Monitoring Board
ECG: Electrocardiograph
ES: effect size
GPH: Global Physical Health; GMH: Global Mental Health.
IAE: Incidence of adverse events
ITT: Intent-to-treat
PP: per-protocol
PROMIS: Patient-Reported Outcomes Measurement Information System
SD: Standard deviation
TCC: Trial coordinating committee
TCM: Traditional Chinese medicine
TSC: Trial steering committee
