# Supplementary material for: Effectiveness of acupuncture for breast cancer related lymphedema: protocol for a single-blind, sham-controlled, randomized, multicenter trial
Source: BMC Complement Altern Med. 2017 Sep 21;17:467. doi: 10.1186/s12906-017-1980-0 (PMC5609040; doi:10.1186/s12906-017-1980-0)
Supplement: Additional file 1: — Block Randomized Sequence. (PDF 83 kb) [file 12906_2017_1980_MOESM1_ESM.pdf]

blocked randomized sequences

block	H1drug	H1placebo	H2drug	H2placebo	H3drug
1	patient1	patient51	patient1	patient31	patient1
2	patient2	patient52	patient2	patient32	patient2
3	patient3	patient53	patient3	patient33	patient3
4	patient4	patient54	patient4	patient34	patient4
5	patient5	patient55	patient5	patient35	patient5
6	patient6	patient56	patient6	patient36	patient6
7	patient7	patient57	patient7	patient37	patient7
8	patient8	patient58	patient8	patient38	patient8
9	patient9	patient59	patient9	patient39	patient9
10	patient10	patient60	patient10	patient40	patient10
11	patient11	patient61	patient11	patient41	patient11
12	patient12	patient62	patient12	patient42	patient12
13	patient13	patient63	patient13	patient43	patient13
14	patient14	patient64	patient14	patient44	patient14
15	patient15	patient65	patient15	patient45	patient15
16	patient16	patient66	patient16	patient46	patient16
17	patient17	patient67	patient17	patient47	patient17
18	patient18	patient68	patient18	patient48	patient18
19	patient19	patient69	patient19	patient49	patient19
20	patient20	patient70	patient20	patient50	patient20
21	patient21	patient71	patient21	patient51	
22	patient22	patient72	patient22	patient52	
23	patient23	patient73	patient23	patient53	
24	patient24	patient74	patient24	patient54	
25	patient25	patient75	patient25	patient55	
26	patient26	patient76	patient26	patient56	
27	patient27	patient77	patient27	patient57	
28	patient28	patient78	patient28	patient58	
29	patient29	patient79	patient29	patient59	
30	patient30	patient80	patient30	patient60	
31	patient31	patient81			
32	patient32	patient82			
33	patient33	patient83			
34	patient34	patient84			
35	patient35	patient85			
36	patient36	patient86			
37	patient37	patient87			
38	patient38	patient88			
39	patient39	patient89			
40	patient40	patient90			
41	patient41	patient91			
42	patient42	patient92			
43	patient43	patient93			
44	patient44	patient94			
45	patient45	patient95			

46	patient46	patient96
47	patient47	patient97
48	patient48	patient98
49	patient49	patient99
50	patient50	patient100

for each center

H3placebo	block	H1drug
patient21	1	0.640854
patient22	2	0.133341
patient23	3	0.573766
patient24	4	0.602637
patient25	5	0.222746
patient26	6	0.340253
patient27	7	0.582022
patient28	8	0.318505
patient29	9	0.818318
patient30	10	0.577358
patient31	11	0.231453
patient32	12	0.028151
patient33	13	0.917096
patient34	14	0.120143
patient35	15	0.84058
patient36	16	0.60043
patient37	17	0.499425
patient38	18	0.827796
patient39	19	0.731476
patient40	20	0.37695
	21	0.437036
	22	0.09581
	23	0.847918
	24	0.161381
	25	0.089586
	26	0.031353
	27	0.951511
	28	0.223968
	29	0.890443
	30	0.972668
	31	0.086006
	32	0.349086
	33	0.133338
	34	0.210987
	35	0.455304
	36	0.24294
	37	0.373177
	38	0.623155
	39	0.787131
	40	0.984235
	41	0.488521
	42	0.970328
	43	0.192582
	44	0.386701
	45	0.771794

46	0.612875
47	0.614744
48	0.035496
49	0.438098
50	0.119862

H1placebo	H2drug	H2placebo	H3drug	H3placebo
0.681207	0.5098091	0.44557243	0.539607	0.369211
0.974534	0.4799152	0.93243627	0.305467	0.364253
0.041139	0.5440746	0.87855879	0.766835	0.178081
0.630917	0.534319	0.75465993	0.159177	0.219025
0.805618	0.9934661	0.62301267	0.57673	0.404981
0.175434	0.4430549	0.66072122	0.668683	0.012413
0.152994	0.7763599	0.66528533	0.663097	0.200991
0.190447	0.0416188	0.64837889	0.102505	0.355893
0.57734	0.129913	0.67430286	0.255981	0.657141
0.503883	0.09593	0.52642964	0.818556	0.869616
0.339943	0.3625454	0.30901708	0.601786	0.425847
0.243818	0.0113642	0.99144436	0.161184	0.202428
0.270875	0.1378983	0.63270326	0.973782	0.478585
0.115145	0.040542	0.82352068	0.643616	0.852982
0.991579	0.7478636	0.7044557	0.342858	0.872994
0.966187	0.3144253	0.57020803	0.596181	0.530187
0.529956	0.9196597	0.17826522	0.860194	0.860685
0.617708	0.3363565	0.1327754	0.965232	0.595584
0.711228	0.7502866	0.05283374	0.023442	0.702329
0.383924	0.3494753	0.2338307	0.167245	0.196533
0.16088	0.9579126	0.5986318		
0.263016	0.7578469	0.17086534		
0.587297	0.5439127	0.71140662		
0.352174	0.9983957	0.41554913		
0.899424	0.7083094	0.31228424		
0.83892	0.6008364	0.72175337		
0.797339	0.2380245	0.80170903		
0.442233	0.7982424	0.48826515		
0.696708	0.2089221	0.89256853		
0.496395	0.515632	0.85828318		
0.770796				
0.97069				
0.278806				
0.960861				
0.410887				
0.442183				
0.573721				
0.673491				
0.784517				
0.407387				
0.398122				
0.084258				
0.109283				
0.665438				
0.122542				

0.812396
0.429035
0.740373
0.477781
0.966486

block	1	2	3	4	5	6
1	2	1	4	5	3	6
2	6	1	3	2	5	4
3	3	6	4	1	2	5
4	3	2	4	1	6	5
5	6	2	1	3	4	5
6	4	5	3	2	1	6
7	4	6	1	2	3	5
8	3	4	6	1	5	2
9	1	4	6	2	5	3
10	3	5	6	4	2	1
11	6	4	3	5	1	2
12	5	2	6	1	4	3
13	2	5	6	3	1	4
14	4	5	6	2	3	1
15	3	1	4	5	6	2
16	2	1	6	4	3	5
17	5	4	1	6	3	2
18	2	3	5	6	1	4
19	2	3	1	5	6	4
20	2	1	3	4	6	5
21	3	4	1	2		
22	4	2	1	3		
23	1	3	4	2		
24	4	3	1	2		
25	4	1	2	3		
26	4	1	3	2		
27	1	3	4	2		
28	4	3	1	2		
29	2	3	4	1		
30	1	4	3	2		
31	2	1				
32	2	1				
33	2	1				
34	2	1				
35	1	2				
36	2	1				
37	2	1				
38	2	1				
39	1	2				
40	1	2				
41	1	2				
42	1	2				
43	1	2				
44	2	1				
45	1	2				

46	2	1
47	1	2
48	2	1
49	2	1
50	2	1
