# Supplementary material for: Effectiveness of acupuncture for breast cancer related lymphedema: protocol for a single-blind, sham-controlled, randomized, multicenter trial
Source: BMC Complement Altern Med. 2017 Sep 21;17:467. doi: 10.1186/s12906-017-1980-0 (PMC5609040; doi:10.1186/s12906-017-1980-0)
Supplement: Additional file 2: — Sample Size Calculation and G Power Manual. (PDF 65 kb) [file 12906_2017_1980_MOESM2_ESM.pdf]

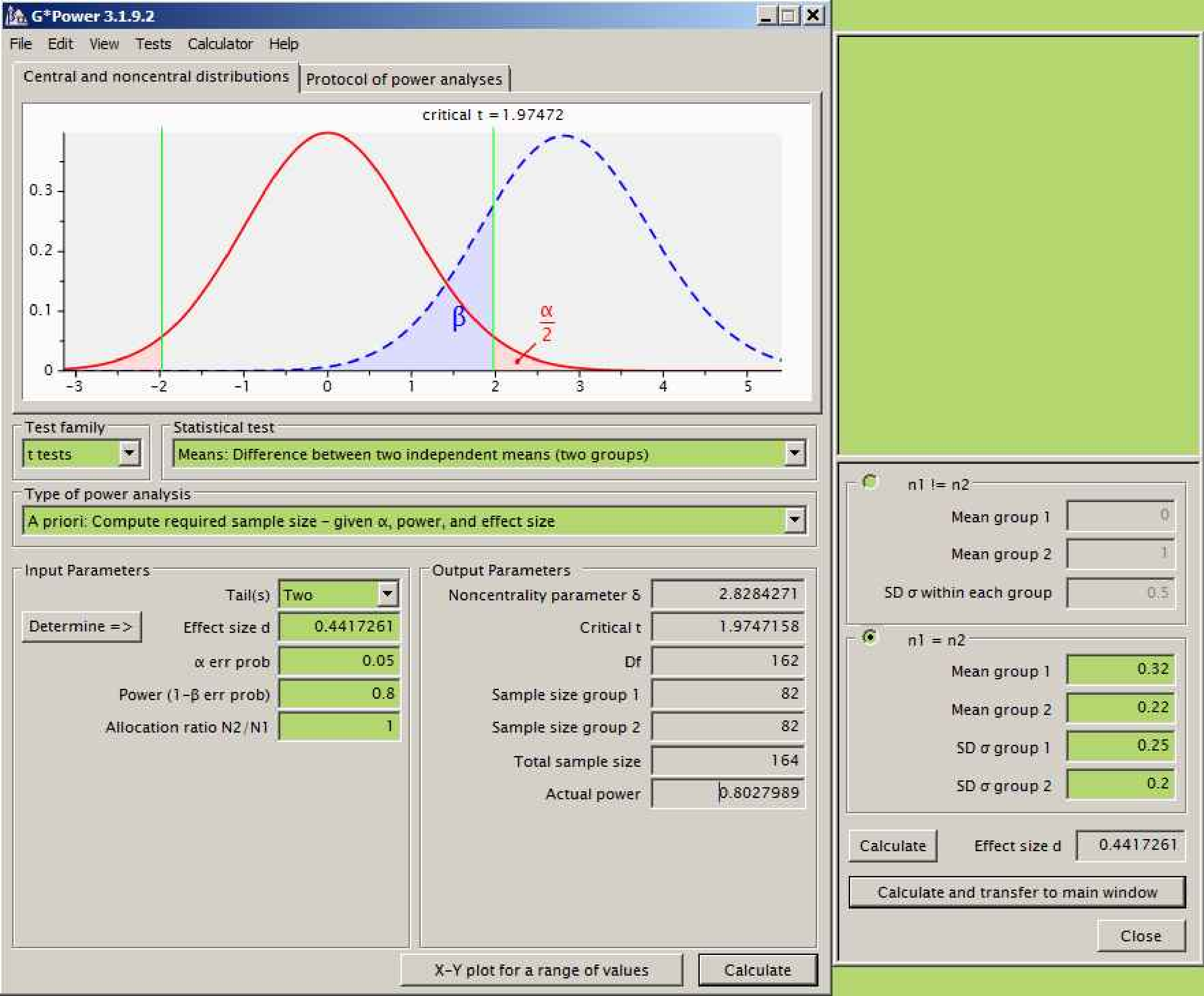
G*Power 3.1.9.2
File Edit View Tests Calculator Help
Central and noncentral distributions
Protocol of power analyses
critical t = 1.97472
0.3
0.2
0.1
0
β
α
2
−3 −2 −1 0 1 2 3 4 5
Test family
t tests
Statistical test
Means: Difference between two independent means (two groups)
Type of power analysis
A priori: Compute required sample size – given α, power, and effect size
Input Parameters
Tail(s) Two
Determine =>
Effect size d 0.4417261
α err prob 0.05
Power (1-β err prob) 0.8
Allocation ratio N2/N1 1
Output Parameters
Noncentrality parameter δ 2.8284271
Critical t 1.9747158
Df 162
Sample size group 1 82
Sample size group 2 82
Total sample size 164
Actual power 0.8027989
X-Y plot for a range of values
Calculate
n1 != n2
Mean group 1 0
Mean group 2 1
SD σ within each group 0.5
n1 = n2
Mean group 1 0.32
Mean group 2 0.22
SD σ group 1 0.25
SD σ group 2 0.2
Calculate
Effect size d 0.4417261
Calculate and transfer to main window
Close
